# Supplementary material for: Natural variation in expression of a plant immune receptor mediates elicitor sensitivity
Source: PLoS One. 2026 Apr 3;21(4):e0343332. doi: 10.1371/journal.pone.0343332 (PMC13048380; doi:10.1371/journal.pone.0343332)
Supplement: Attachment — Submitted filename: Response letter_final.docx [file pone.0343332.s003.docx]

**Response to editor and reviewers re: PONE-D-25-47849 (“Natural variation in expression of a plant immune receptor mediates elicitor sensitivity”)**

Dear Dr. Chang,

Thank you very much for the handling and evaluation of our manuscript for consideration at PLOS One.

Below is the original correspondence from Oct 9 2025 (colored black) alongside our responses (colored blue). We hope that the changes we describe will address the issues raised in the reviews and evaluations.

Thank you for submitting your manuscript to PLOS ONE. After careful consideration, both reviewers gave positive feedback on this study, and we feel a minor revision and clarification would be needed before acceptance. Therefore, we invite you to submit a revised version of the manuscript that addresses the points raised during the review process.

We thank the editor and reviewers for the comments. Please find our responses below.

Reviewer #1: The manuscript by Behnken, George and colleagues reports an extensive genetic analysis of INR variation in common bean landraces. This work builds on the Steinbrenner group’s long-standing establishment of the ligand-receptor system as a model for herbivore-plant interactions. Focusing on an “insensitive” phenotype to the ligand (In11) treatment with the measurement of ethylene response, they identified promoter variation underlying differences in ethylene responses. They further demonstrated a significant correlation between natural variation in the promoter sequences (and thus haplotypes) and In11-sensitivity, with functional confirmation of promoter activities in the heterologous N. benthamiana system. Attempts to build a strong causative relationship have been made by generating NILs carrying the inr-2 allele. Notably, they found that the inr-2 carriers, even with low INR expression, can still confer resistance comparable to wild-type carriers. The authors provide an insightful explanation of the complex relationship between insensitivity and resistance. Their interpretation indeed deepens our understanding of how these promoter dynamics might play out in nature. Their discussion on the distinct mode evolutionary trajectories of promoter vs. genic sequences is pertinent to the new finding in this manuscript. This highlights the composite effector of various regulatory elements (e.g. TF binding sites) in finely regulating receptor function under the inherently stochastic pressures of herbivore-plant interactions.
This is an excellent and timely contribution. Rather than presenting artificially perfect datasets, it captures the complexity of natural variation and reveals the limitations of laboratory assays in fully recapitulating the forces shaping immune receptor regulation. Thus, this work provides an important gateway to improve the assay conditions to better reflect the conditions relevant for shaping the natural variation in future work.

We thank the reviewer for the comments on the manuscript and data interpretations.

Here, I provide minor comments that could be used for textual edits.
• L80: Is it necessary to include (LRR-RLP) as it is in the parenthesis?

Thank you, we changed this in the revised manuscript.

• L191: The subheading can be more direct in describing the content. It is not super clear what “different” herbivore resistance means.

Thank you for helping us clarify. The heading now reads “In11 insensitivity in low INR expression lines does not correspond with reduced herbivore resistance”

• Regarding the NIL-inr-2-line build-up, would the authors consider the possibility of the recurrent parent G19833 line carrying a genetic modifier (e.g. enhancing INR’s capability of conferring herbivore resistance)? It seems like the pollination/crossing of inr-2 (or other insensitive lines) seems challenging, but if there are any other NILs in different backgrounds available, the result can be reaffirmed. May the authors consider this notion as a discussion point.

Thank you for the alternative interpretation. We summarized this possibility in the discussion:

“Third, other genetic modifiers in the recurrent parent background (variety G19833) may boost strength of In11-induced responses, overcoming the effect of *INR* expression strength. Additional NILs developed in alternate backgrounds would be needed to disentangle the effect of *inr-2* from its genetic background.”

• Some of the past experiments and results were condensed (e.g. L197-202). Without the knowledge of the study system, it can be a bit hard to follow. The authors can expand such parts to enhance readability.

Thank you for helping us clarify. We have made extensive edits to the results section, including L196-207 (now lines 207-216), to better explain the study system. Please see the Track Changes document.

Reviewer #2: Summary
Plant immune systems rely on pattern recognition receptors to detect diverse enemy-associated molecular patterns. The role of transcriptional regulation of these receptors is poorly understood. When functionally characterizing a genetic polymorphism for sensitivity to the herbivore-derived elicitor Inceptin in common bean, the authors serendipitously discovered that cis-regulatory variation underlies this variation. They elegantly integrated methods in plant molecular biology and classical genetics to convincingly demonstrate that cis-regulatory variation is due to promoter variation upstream of the receptor, and they found a surprising result that the organism-level immune response initiated by this receptor can be mounted to blunt herbivory even in genotypes with alleles causing the receptor to be expressed at a low level. This has fascinating implications for how plants allocate resources between growth and immunity/defense, is of broad interest, and will spur future investigations.
Overall, the manuscript is very well-written and results are presented clearly. Integration with the broader literature is deep and thoughtful: the authors put the work in context of other immune receptors to identify knowledge gaps regarding functional variation in pattern recognition receptors and the interactions between herbivores and the plant immune system.

We thank the reviewer for the positive comments on the manuscript.

Major Comments
None.

Minor Comments
Generally, this is written to be accessible to a broad audience. There are a couple lapses, however. For Figure 2A and related text: the experimental summaries in the results section, the figure captions, and the brief figure labels together did not clearly convey the experiment. Additionally, acronyms from molecular genetics sometimes are used without being defined (e.g., TSS). Acronyms are also used a bit heavily in general, especially in figure captions (e.g., RLU and TF in Fig. 2). Ideally, figure captions should be understandable without searching the text for definitions of acronyms. Although these acronyms may be common in certain fields, I doubt most readers will know all of them. Some extra attention to clarity of descriptions and use of acronyms would demand less effort from the reader and thus allow easier focus on the science being presented.

Thanks for the encouragement to further clarify figure captions. We have paid special attention to each main Figure caption, especially relating to the specific comments below relating to Fig. 2 clarity.

Fig. 1D: It might be helpful to use a distance-based clustering approach to order the accessions (keeping the same color-coding). You could either only show the tree and put the distance matrix in the supplement, or display the tree lining up with the distance matrix. At a glance, it does look like some responsive accessions could cluster in a group with the non-responsive accessions, and it would be nice to get this nuance.

We appreciate this suggestion for more rigorous clustering analysis. We added a new Fig. 1D as shown below.

Fig. 2A & L124-127: The text describes generation of F2’s from a biparental cross, which I assumed to mean standard RILs derived from that cross (i.e., as are used in QTL mapping), and references Fig. 2A. But Fig. 2A’s caption refers to something more specific: back-crossed individuals. Fig. 2A itself does not clarify this to me. I presume F2’s can be heterozygous or homozygous for either allele, so I’m not sure why the two genotypes are “G19833/-” (what does – mean?) and “PI638858” (are these only homozygous plants)? Clarification is needed.

We apologize for the lack of clarity. We re-wrote this part of the caption as **A)** In11 sensitivity corresponds with INR locus genotype in F2 plants. Ethylene production was measured after scratch-wounding and treatment with H_2_O or 1 μM In11 peptide, with ratio greater than 1 indicating induced response. n=11 F2 plants were tested and are displayed according to genotype at position 7,408,876, with plants homozygous or heterozygous for G19833 allele (G19833/-) or homozygous for PI 638858 allele (PI 638858).

L62: Recent work indicates that ACD6 allelic effects are highly environmentally-dependent and may be dramatically altered (to the point of not being observable) in field conditions. This environmental dependence is worth mentioning here. <https://journals.plos.org/plosbiology/article?id=10.1371/journal.pbio.3003237>

Thanks for the helpful reference. We cited it at Line 315:

“ Finally, defense phenotypes observed at single timepoints in growth chambers may not correspond with phenotypes in longer timecourses and more complex environmental conditions. In a recent example of this theme, natural alleles of ACD6 conferring striking differences in autoimmunity and growth phenotypes in growth chambers did not show differences in field settings (48).”

L102: Table S2 lists P > 0.05 for 11 species, not 8. Which of the three responsive species with P > 0.05 is not indicated in the corresponding figure? I believe the discrepancy is PI638862.
Thanks for the push to double check on this. There are actually 12 varieties with P > 0.05 as listed in the table, which now fully correspond with Fig 1A – there are 8 “Unresponsive” varieties, as well as 4 additional varieties where there was no statistically significant difference (“n.s.”) but where the In11/H2O ratio was >1.9. We labeled these latter 4 as “Responsive”. We have double checked all tables and figures.

L143: I don’t understand the phrasing “the first 800 bp of 1.8 kb from TSS (0 bp = TSS)”. What is the significance of 1.8 kb? When I got to the end of the caption, I realized that perhaps 1.8 kb upstream of the TSS was analyzed for TF binding sites, but none were found more than 800 bp from the TSS? In that case, it would be clearer to simply say this rather than break it up into two non-continuous parts of the caption.
L144-145: “TF families…” is vague. Is the intended message something like, “TF families whose predicted binding sites contain SNPs and deletions are annotated”?

Thanks for asking for clarification. We rewrote the Fig. 2D caption addressing the L143 and L144-145 points. It now reads as follows

“Alignment and Transcription Factor (TF) binding prediction of SNPs in TF binding sites upstream of the INR Translation Start Site (TSS) (0 bp = TSS). SNPs were identified from INR promoters used in luciferase assays. SNPs and deletions are indicated by red lines relative to the sequence of pINR^PI 638852^. Only SNPs predicted to alter a TF binding site are shown: 1 - HD-Zip, 2 - Dof, 3 - MIKC-MADS, 4 - ERF and MYB, 5 - bZIP, bHLH, and Nin-like, 6 - MYB. TF binding was predicted with PlantRegMap parameterized with the Arabidopsis thaliana reference dataset (33). Full analysis of SNPs, including those with no predicted TF binding site, is shown in S4A Fig.”

L298: Was fertilizer used?

Thank you for asking for that detail. We added those details as outlined below

“Starts were fertilized once with 50g Jack’s Professional 20-20-20 General Purpose mixed in with 4L of water… Seedlings were fertilized every other week starting at 3 weeks old with 25g Jack’s Professional 20-20-20 General Purpose mixed with 4L water.”

L316: Given that ~15% of sites had less than 4x read depth, and low coverage sites were filtered … were there ever cases where an accession that was investigated for INR promoter SNPs did not have sufficient coverage to call the genotype at one or more SNPs? If so, it would be important to note that the genotype is uncalled rather than wildtype in the genotype plots.

Thank you, we now note how we treat coverage in the materials and methods: “We have marked SNP positions where there was lack of coverage in S2B Fig.”

L345-367: Please indicate antibiotics used for selection or growth in all cases.

Thank you, we added details in all cases in the revised manuscript.

L67: EFR is not defined

Thank you, we changed this in the revised manuscript.

L80: Why is “LRR-RLP” in parentheses?

Changed

L94: For consistency, should have a dash (“In11-unresponsive”)

Changed

L95: How many In11-unresponsive accessions were identified among the 91 screened?

Thanks for helping us clarify. Only two accessions were originally identified among the 91.
